# Choosing the Right Tool for Genetic Engineering: Clinical Lessons from Chimeric Antigen Receptor-T Cells

**DOI:** 10.1089/hum.2021.173

**Published:** 2021-10-18

**Authors:** Melita Irving, Evripidis Lanitis, Denis Migliorini, Zoltán Ivics, Sonia Guedan

**Affiliations:** ^1^ Department of Oncology, Ludwig Institute for Cancer Research Lausanne, Lausanne University Hospital and University of Lausanne, Lausanne, Switzerland.; ^2^ Department of Oncology, Geneva University Hospitals, Geneva, Switzerland.; ^3^ Center for Translational Research in Onco-Hematology, University of Geneva, Geneva, Switzerland.; ^4^ Swiss Cancer Center Léman, Geneva and Lausanne, Switzerland.; ^5^ Transposition and Genome Engineering, Division of Medical Biotechnology, Paul Ehrlich Institute, Langen, Germany.; ^6^ Department of Hematology and Oncology, Hospital Clinic, August Pi i Sunyer Biomedical Research Institute (IDIBAPS), Barcelona, Spain.

**Keywords:** T cell engineering, CAR-T cells, viral vectors, nonviral vectors, genome editing

## Abstract

T cell modification with genes that encode chimeric antigen receptors (CAR-T cells) has shown tremendous promise for the treatment of B cell malignancies. The successful translation of CAR-T cell therapy to other tumor types, including solid tumors, is the next big challenge. As the field advances from second- to next-generation CAR-T cells comprising multiple genetic modifications, more sophisticated methods and tools to engineer T cells are being developed. Viral vectors, especially γ-retroviruses and lentiviruses, are traditionally used for CAR-T cell engineering due to their high transduction efficiency. However, limited genetic cargo, high costs of production under good manufacturing practice (GMP) conditions, and the high regulatory demands are obstacles for widespread clinical translation. To overcome these limitations, different nonviral approaches are being explored at a preclinical or clinical level, including transposon/transposase systems and mRNA electroporation and nonintegrating DNA nanovectors. Genome editing tools that allow efficient knockout of particular genes and/or site-directed integration of the CAR and/or other transgenes into the genome are also being evaluated for CAR-T cell engineering. In this review, we discuss the development of viral and nonviral vectors used to generate CAR-T cells, focusing on their advantages and limitations. We also discuss the lessons learned from clinical trials using the different genetic engineering tools, with special focus on safety and efficacy.

## Introduction

In recent years, immunotherapy, including immune check-point blockade and adoptive cell therapy (ACT), has become the fourth pillar of cancer treatment along with surgery, chemotherapy, and radiotherapy.^1^ Durable and complete responses in patients with advanced hematological malignancies have been achieved in multiple independent treatment centers upon ACT of chimeric antigen receptor (CAR)-modified T cells (reviewed in June and Sadelain^2^).

These unprecedented outcomes, including complete responses in >80–90% of adults and children with relapsed/refractory acute lymphoblastic leukemia (ALL),^3^ led to rapid Food and Drug Administration (FDA) regulatory approval of the CD19-directed CAR-T cell products Kymriah and Yescarta in the United States in 2017, and in Europe and other parts of the world the following year, to treat ALL and diffuse large B cell lymphoma. There are now five CAR-T cell therapies approved for the treatment of B cell malignancies targeting CD19 and B cell maturation antigen (BCMA).

CARs are hybrid transmembrane receptors comprising an extracellular tumor-antigen binding moiety, typically in the form a single-chain variable fragment (scFv), linked to signaling endodomains allowing T cell activation and effector functions upon target engagement. While first-generation CARs include the endodomain of CD3 zeta only (for signal 1 of T cell activation), second- and third-generation CARs further comprise one or more costimulatory endodomains (signal 2), respectively, usually derived from CD28 or 4–1BB molecules.^4^

Most CARs currently used in the clinic are second generation and despite that clinical data since 2010 indicate their potential to be curative against advanced leukemia, lymphoma, and multiple myeloma, there are many barriers that limit their efficacy against other liquid tumor types, as well as epithelial-derived solid tumors, which represent the vast majority of cancers.

CAR-T cells, for example, oftentimes do not sufficiently engraft and persist, including for most chronic lymphocytic leukemia (CLL) patients, and this is a property that appears critical to a positive clinical outcome.^5^ In other instances, it can be difficult to collect sufficient T cell numbers for manufacturing purposes due to underlying disease, age, and prior therapies,^6^ or the patient status is so advanced that they are no longer able to receive the infusion by the time the autologous CAR-T cell product is ready. These observations have led to important efforts to develop allogeneic “off-the-shelf” CAR-T cell products.^7^

With respect to solid tumors, critical barriers include a paucity of homogeneously expressed target tumor antigens that are not found on critical healthy tissue(s), as well as limited T cell homing and migration into the tumor bed. In addition, a range of inhibitory conditions in the tumor microenvironment (TME) can lead to T cell exhaustion or anergy, including chronic antigen stimulation, insufficient costimulation, low pH, limited oxygen and nutrients, as well as exposure to toxic metabolites and a range of suppressive molecules and receptors such as TGFb and PD-L1, respectively.^4^

It is now widely held that personalized combinatorial treatment and/or coengineering approaches are needed to extend and improve clinical responses in cancer patients to CAR therapy.^8^ Coengineered CAR-T cells are referred to as next-generation or fourth-generation CAR-T cells, or more specifically as TRUCKS (T cells redirected for universal cytokine-mediated killing) if the additional gene cargo is a cytokine.^4^ To increase treatment safety, important research efforts are also focused on developing remote control CAR designs, including ON- and STOP-switches, as well as to introduce logic gates into the cells to allow inducible behavior based on TME cues (reviewed in Guedan et al.^9^). Such strategies may be particularly critical as next-generation CAR-T cells, which may have a greater risk for toxicity, reach the clinic.

**Figure 1.**
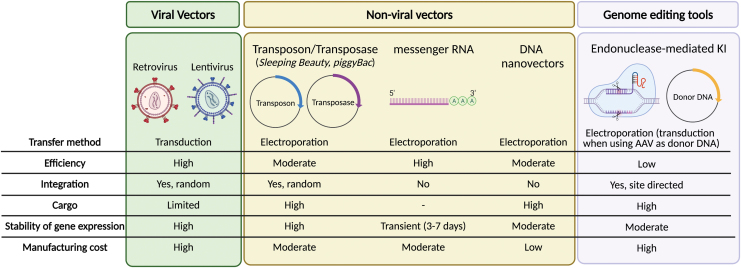
Summary of the current vectors used to genetically modify T cells to express CARs. Color images are available online.

In addition to personalized coengineering strategies and improved receptor design, choice of genetic engineering tool(s) is a critical parameter for the clinical translation of CAR-T cell products. Important considerations in the choice of engineering tool are efficiency and stability of transgene expression (unless transient expression is desired), genetic cargo capacity, safety (including low vector-related genotoxicity), and possibility to rapidly scale-up production for clinical translation at low cost. Here, we review the abovementioned engineering tools for producing CAR- and next-generation CAR-T cells, outline the advantages and limitations each of them offer based on results obtained in preclinical and clinical trials (Fig. 1), and provide insight into where the field is headed.

## Viral Vectors: γ-Retrovirus Versus Lentivirus

γ-Retroviral and lentiviral vectors are commonly used for CAR-T cell generation due to their ability to achieve high rates of transduction and long-term stable transgene expression. To date, all CAR-T cell therapies approved for commercialization use γ-retroviruses or lentiviruses for genetic engineering.

### Biology and development of viral vectors

The viral family Retroviridae includes seven members, with two of them, the γ-retroviruses and the lentiviruses, being widely used for genetic engineering of T cells.^10^ Retroviruses consist of lipid-enveloped particles comprising a single-stranded diploid RNA genome (two copies of RNA genome per virus particle) and an RNA-dependent DNA polymerase (reverse transcriptase). The early steps of retroviral replication involve reverse transcription of the viral RNA genome into cDNA, which then integrates into the host chromosomal DNA.

The integration reaction requires specific sequences at the long terminal repeat (LTR; sequences that define the boundaries of the viral genome) and a viral-encoded integrase. The fact that retroviruses can integrate their genome into the DNA of the host cell, ensuring long-term expression of the viral genes, makes them particularly suited as gene transfer vectors.^11^

Typically, viral vectors used to generate CAR-T cells derive from the Moloney murine leukemia virus (Mo-MLV) or the human immunodeficiency virus (HIV).^12^ To generate a vector, the viral coding sequences are removed from the viral backbone and replaced by the transgene of interest, such as the CAR. In this way, the transgene sequence is flanked by LTR sequences required for the integration of the transgene. The U3 region of the LTR is also necessary for retroviral RNA transcription under the control of endogenous enhancer/promoter sequences.

All of the basic genes required to package the recombinant viral genome, including the *gag*, *pol*, and *env* genes, are provided in *trans* for viral production in packaging cell lines; *gag* encodes for capsid proteins, *pol* encodes for enzymes needed for reverse transcription and integration into the host cell genome, and *env* encodes for the viral envelope glycoprotein. While both retroviruses and lentiviruses require *gag*, *pol*, and *env* genes for packaging, they use slightly different isoforms. In the case of lentiviral vectors, the *rev* gene is also required in *trans* to enhance the nuclear export and expression of gag-pol transcripts. The Psi packaging element (ψ) allows the transcripts to be packaged into virions with high efficiency.

When generating γ-retroviral or lentiviral vectors, there is a theoretical risk for recombination events that could result in the generation of replication-competent retrovirus or lentivirus (RCR/L) during vector manufacturing or in patients after treatment. To decrease this risk, the genes required for vector production have been split across different plasmids.

Another strategy to increase safety is the introduction of deletions in the U3 region of the 3′LTR, a region responsible for the promoter/enhancer activity of the LTR. The deletion in the 3′LTR is transferred to the 5′LTR during reverse transcription, resulting in transcriptional inactivation of the provirus and rendering the virus “self-inactivating” (SIN) after integration.^13^ Then, an exogenous promoter is placed immediately upstream of the transgene cDNA. SIN vectors have been constructed based on MLV or lentiviruses, and most of the lentiviral vectors used in clinical applications are third-generation SIN vectors.^12^ However, the use of retroviral SIN vectors in the clinic is limited because deletions in the 3′LTR prevent the generation of high-titer vectors using packaging cell lines commonly used for retrovirus manufacturing under good manufacturing practice (GMP) conditions.

### Vector tropism: pseudotyping viral vectors to transduce T cells

The ability of the viral vector to transduce different host cell types, known as tropism, is dictated by the env protein.^14^ Modification of the virus glycoprotein (pseudotyping) can alter and improve the tropism of viral vectors for different primary cell types. Ecotropic virus infects only rodent cells; amphotropic virus can transduce a broad range of mammalian cells, while pantropic virus can infect cells of any species. Mo-MLV is an ecotropic virus, and therefore, vectors derived from this virus are the vectors of choice to genetically modify murine T cells.^15^ To achieve transduction of human T cells, γ-retroviral vectors can be pseudotyped to express the envelope of the amphotropic MLV, the gibbon ape leukemia virus, or the feline endogenous virus RD114.

The vesicular stomatitis virus glycoprotein (VSV-G) is commonly used for pseudotyping lentiviral vectors. VSV-G recognizes the ubiquitously expressed low-density lipoprotein (LDL) receptor, allowing the transduction of a wide range of cells (pantropic). Of note, the LDL receptor is expressed at low densities on resting T cells, but upregulated upon T cell activation.^16^ Thus, genetic modification of T cells with lentiviral vectors is commonly performed after *in vitro* T cell activation. A limitation of using the VSV-G is that it is toxic to packaging cell lines, and therefore, vectors pseudotyped with VSV-G can only be packaged by transiently transfecting the *VSV-G* gene into the producer cells.

### Driving high and stable transgene expression

When using γ-retroviral vectors, the enhancer/promoter sequences located in the U3 region of the LTR can drive the expression of the transgene of interest. However, SIN retroviral and lentiviral vectors with deletions in the U3 region of the LTR require an exogenous promoter to drive the expression of the transgene. The cytomegalovirus promoter can drive robust transgene expression in most cell lines that are actively dividing but show a greater variation of expression when used in T cells. The elongation factor 1-α (EF1-α) promoter drives higher levels of transgene expression over time in T cells and is therefore the promoter of choice for most CAR-T cell products.^17^ Stable CAR expression is typically obtained with the incorporation of the post-translational regulatory element of the woodchuck hepatitis virus (WPRE) in the 3′UTR.^18^

### Viral vectors used in the clinic

The first clinical trial testing T cells genetically engineered to express an exogenous transgene after transduction with retroviral vectors was concluded more than three decades ago.^19^ Since then, hundreds of patients have been successfully treated with genetically engineered T cells using viral vectors. Long-term follow-up of clinical studies supports the safety and efficacy of this approach.^20,21^

Of the five FDA-approved CAR-T cell products, two of them comprise γ-retroviral vectors (Yescarta^®^ and Tecartus^®^) and three others are lentiviral vectors (Kymriah^®^, Breyanzi^®^, and Abecma^®^). Yescarta and Tecartus use the murine stem cell virus (MSCV)-based splice-gag γ-retroviral vector (MSGV1), a vector derived from the MSCV, containing an extended *gag* region and a Kozak sequence to enhance translational efficiency.^22^ In this vector, CAR expression is driven by the promoter located in the MSCV 5′LTR. This LTR promoter has previously shown to promote high levels of gene expression in a variety of cell types, including primary hematopoietic cells and T cells. Kymriah, Breyanzi, and Abecma are genetically engineered with third-generation SIN lentiviral vectors derived from the HIV, with EF1-α driving the expression of the CAR.^23^

As of now, there is no preclinical or clinical evidence suggesting that the use of one vector is significantly better than the other for generating engineered T cells. Additional differences in the design of CAR-T cell clinical trials, including the method of T cell stimulation, the manufacturing process, the CAR design, or the T cell subsets used, may influence the clinical outcome and preclude a better understanding of the role of the gene delivery system into T cells.

### Clinical implications of vector integration in the T cell genome

A key question that is gaining attention in the field is whether CAR integration in the T cell genome can affect the safety and efficacy of CAR-T cell therapy. Genomic integration of retroviral and lentiviral vectors can potentially result in oncogenic transformation, clonal expansion, or heterogenous levels of CAR expression (Table 1). While the integration of retroviral vectors is uncontrolled, it is not a random process as some parts of the genome are favored. Each retrovirus class exhibits a unique and preferred pattern of integration within the host genomes, which is reproduced by the gene transfer vectors derived thereof.

**Table 1.** Integration and risk of T cell transformation with clinically available gene transfer vectors

	Preferential Insertion	Transformation of Hematopoietic Progenitors	T Cell Transformation	Clonal Expansion/Dominance	Reported Patients Treated with Engineered T Cells/Follow-Up
*Retroviral vectors*	Near transcriptional start sites and CpG islands	Leukemia, lymphoma, and myelodysplastic syndrome observed in gene therapy trials	Not observed	Not observed	Hundreds of patients/>30 years
*SIN Lentiviral vectors*	Active transcription units	Not observed	Not observed	Yes	Hundreds of patients/>20 years
*Sleeping Beauty*	Random distribution	Not observed	Not observed	Not observed	Tens of patients/>10 years
*PiggyBag*	Near transcriptional start sites, CpG islands, and DNaseI hypersensitive sites	Not observed	Lymphoma (two patients)	Not observed	Tens of patients/>5 years
*Endonuclease enzymes/donor DNA*	Site directed	Not observed	Not observed	Not observed	Tens of patients/>5 years

SIN, self-inactivating.

γ-Retroviruses exhibit preferential insertion near transcriptional start sites and CpG islands, including enhancers and promoters (reviewed in Cavazza et al.^24^). The retroviral enhancer/promoters located in the LTR are particularly promiscuous and can activate various types of promoters in different configurations. If integration occurs near a proto-oncogene, this could result in oncogenic transformation.

The use of MLV in clinical trials targeting hematopoietic stem cells for the treatment of inherited immunodeficiencies resulted in insertional mutagenesis that led to leukemia/lymphoma in a significant number of patients.^24,25^ Since then, the safety of viral vectors has been carefully monitored. However, no evidence of RCR or oncogenic transformation has been observed after treatment of hundreds of patients with T cells genetically modified with retroviral vectors, including treatment with Yescarta.^19,20^ This is in line with the observation that mature lymphocytes are less prone to transformation due to proapoptotic and epigenetic mechanisms that potentially prevent clonal outgrowth.^26^

Contrary to retroviruses, lentiviruses show preferential insertion in the introns of transcriptionally active genes, and therefore, their risk of insertional oncogenesis is lower. Furthermore, third-generation SIN lentiviral vectors containing cellular promoters derived from human genes (such as EF-1a and phosphoglycerate kinase) have been further optimized to reduce this possibility.^27^ RCL or transformation has not been reported in patients treated with lentivirus-modified T cells, with accumulated safety data available in several 100 study subjects.^21^

However, recent reports indicate that the site of lentiviral vector-mediated CAR-integration within the T cell genome can affect T cell proliferation,^28^ influencing the therapeutic outcome. In this regard, a recent study described an unusual case of a profound expansion of a single CD19-CAR-T cell clone that led to a complete response in a patient with leukemia. Characterization of this T lymphocyte population indicated that the CAR transgene had integrated into the *TET2* locus in a patient with a hypomorphic mutation in the second *TET2* allele.^28^ A second study identified several integration sites in the *TET2* locus, but no signs of clonal expansion were observed, indicating that biallelic disruption of the *TET2* locus may be required for clonal expansion.^29^

Finally, a larger follow-up study using data from CLL patients undergoing CART19 therapy showed that the distributions of lentiviral vector integration sites in CAR-T cells could distinguish patients showing positive clinical responses from those showing limited or no responses.^30^

Altogether, these studies highlight the importance of genetic engineering and the need to better understand the role of vector integration on the therapeutic window of T cell therapies. In this direction, Wang *et al.* recently reported the development of a method called EpiVIA for the joint profiling of the chromatin accessibility and lentiviral integration site analysis at the population and single-cell levels.^31^

In summary, CAR-T cells generated using γ-retroviral or lentiviral vectors have shown impressive antitumor responses, and secondary effects related to the presence of RCL or T cell transformation have not been observed. A main drawback of these vectors is the costs associated with clinical-grade manufacturing and safety-monitoring methods. In addition, their limited cargo capacity restricts the number of additional transgenes that can be expressed by CAR-T cells. To overcome these limitations, other gene transfer methods are being explored as an alternative to viral vectors, including transposon/transposase systems, mRNA electroporation, and genome editing tools. Figure 1 summarizes the main advantages and disadvantages of each of these approaches.

## Virus-Free Therapeutic Gene Transfer

### Transposon systems: *Sleeping Beauty and piggyBac*

Transposons are nature's simplest gene delivery vehicles that can be harnessed as highly effective tools for versatile applications in genetic engineering, including gene therapy (reviewed in Ivics et al. and Amberger and Ivics^32,33^). DNA transposons are genetic elements with the ability to change their positions within the genome.^34^ In nature, these elements exist as mobile (“jumping”) units of DNA containing a transposase gene flanked by terminal inverted repeats (TIRs) that carry transposase binding sites. Importantly, it is possible to separate the two functional components of the transposon (the TIRs and the transposase) in the form of bicomponent vector systems.

**Figure 2.**
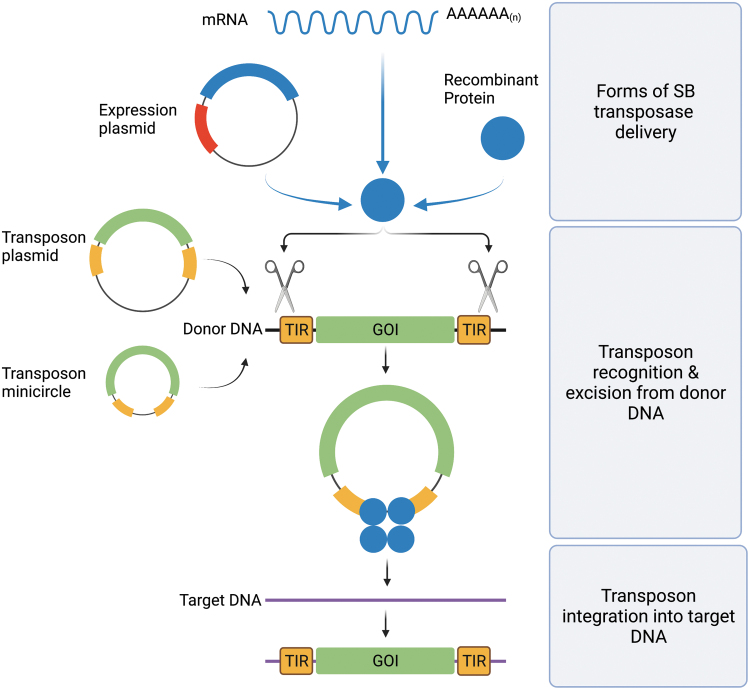
Schematic overview of gene delivery with *SB* transposition. The SB transposase is introduced into a cell in the form of DNA (expression plasmid), mRNA, or recombinant protein along with donor DNA in which the transposon to be mobilized is located. Donor DNA can be vectorized as plasmids or minicircles. After binding within the TIRs of the transposon (TIRs, *yellow rectangles*) flanking a GOI (GOI, *green rectangle*), SB transposase (*blue circles*) performs the excision of the transposon from the donor DNA (*black strand*) and integrates it into a site in the genomic target DNA (*purple strand*). GOI, gene of interest; SB, Sleeping Beauty; TIR, terminal inverted repeat. Color images are available online.

Transposon-based vectors enable incorporation of virtually any DNA sequence of interest between the transposon TIRs and mobilization by *trans*-supplementing the transposase (Fig. 2). In the transposition process, the transposase enzyme mediates the excision of the element from the donor vector, followed by integration of the transposon into a chromosomal locus (Fig. 2). This feature uniquely positions transposons as nonviral gene delivery systems that unite the favorable characteristics of integrating viral vectors (*i.e.*, stable chromosomal integration and long-lasting transgene expression) with those of nonviral delivery systems (*i.e.*, lower immunogenicity, enhanced safety profile, and reduced costs of GMP manufacture).

Based on ancient, inactive transposon sequences isolated from fish genomes, an active transposon was reconstructed, and named *Sleeping Beauty* (SB).^35^ SB was the first transposon ever shown capable of efficient transposition in vertebrate cells, thereby enabling new avenues for genetic engineering, including gene therapy (recently reviewed in Amberger and Ivics^33^).

The advantages of SB transposon-based gene delivery include the following: (1) permanent genomic insertion of transgene cassettes can lead to sustained and efficient transgene expression, (2) in contrast to nonintegrating viral vectors whose repeated *in vivo* administration can provoke immune responses against vector-encoded proteins, only a single administration of SB vectors is required resulting in diminished immunogenicity *in vivo*, (3) as opposed to adeno-associated virus (AAV)- and retroviral vectors that undergo a severe loss of titer beyond a certain vector size, SB vectors have no strict limitation with respect to the size of genetic cargo, (4) superior biosafety profile associated with a lack of biased integration into transcription units and transcriptional regulatory regions of genes, and (5) in contrast to viral vectors, transposon vectors can be maintained and propagated as plasmid DNA, which makes them simple and inexpensive to manufacture, an important consideration for implementation and scale-up in clinical practice (recently reviewed in Amberger and Ivics^33^).

Another transposon system that has been used with success to engineer human cells is called *piggyBac* (PB). Many of the advantages associated with the application of the SB system detailed above are also applicable to PB. However, PB displays an MLV γ-retrovirus-like genome-wide integration profile (including an enrichment of insertions into transcriptional start sites of genes), raising safety concerns^36^ (Table 1).

#### Clinical results with CAR-T cells genetically modified with transposition systems

SB transposition-based nonviral gene delivery has an outstanding potential to provide innovative and potentially curative treatments for an array of monogenetic disorders and cancer (recently reviewed in Amberger and Ivics^33^). SB successfully entered the clinical stage in 2011 with two clinical trials as the first nonviral vector being used to generate CD19-specific CAR-T cells for adjuvant immunotherapy targeting minimal residual disease of non-Hodgkin's lymphoma (NHL) and ALL following hematopoietic stem cell transplantation (HSCT).^37^ Here, SB was successfully used to shuttle a second-generation, CD19-specific CAR cassette in a classic double-plasmid delivery setting.^38^

The trials resulted in 30-month progression-free rates of 83% for patients who received autologous HSCT and 12-month progression-free rates of 53% for patients who received allogenic HSCT. Overall survival rates were 100% for the autologous and 63% for the allogenic HSCT group. Neither transgene integration hotspots nor acute or late toxicities or exacerbation of graft-versus-host disease (GVHD) was observed. These pilot studies established safety and illustrated the potential to use SB in CAR-T cell engineering.

Furthermore, the CARAMBA clinical trial (Phase-I/IIA; EudraCT: 2019-001264-30) investigates the feasibility, safety, and antimyeloma efficacy of autologous SLAMF7 CAR-T cells. CARAMBA is the first clinical trial relying on SB technology for CAR-T cell manufacturing in Europe, and the first clinical trial that uses advanced SB technology (hyperactive SB100X transposase encoded as synthetic mRNA in conjunction with CAR transposon supplied as minicircle vectors) worldwide.^39^

In addition, SB has been used to generate allogeneic “off-the-shelf” CAR-Natural Killer (NK) cells that have shown encouraging results against solid tumors.^40^ Cytokine-induced killer (CIK) cells, effector lymphocytes displaying a mixed T and NK phenotype with nonhuman leukocyte antigen (HLA)-restricted cytotoxicity and minimal alloreactivity, have also been generated with SB to target CD19, CD123, and CD33.^41,42^ Importantly, it has been recently reported that allogeneic, donor-derived CD19 CAR-CIK cells engineered with the SB system demonstrated high expansion, low toxicity, and complete remission in patients with relapsed and refractory ALL in a Phase I/II trial.^43^ Finally, SB was shown to be successful for T cell receptor (TCR) engineering,^44,45^ a strategy that enables to broaden the spectrum of targets as the major histocompatibility complex-TCR interaction also permits recognition of epitopes from intracellular proteins. There are currently a total of 14 active clinical trials in gene therapy making use of SB gene transfer technology (recently reviewed in Amberger and Ivics^33^).

A first-in-human Phase-I study conducted in Australia (The CARTELL Study, NHMRC identifier: 1102172) introduced PB into the clinics in 2016, used to manufacture CD19-specific CAR-T cells infused to patients suffering from relapsed/refractory CD19^+^ malignancies, namely B cell ALL and NHL. Preliminary reports suggest similar results as expected with viral vector-generated CD19 CAR-T cells.^46^ Two additional clinical trials with centers in Japan (UMIN Clinical Trials registry ID: UMIN000030984) and China (clinicaltrials.gov ID: NCT04289220) are currently being conducted/planned, making use of the PB system as well, to manufacture CD19-specific CAR-T cells to treat CD19^+^ B cell malignancies.

Furthermore, expanding on CAR-T cell targets, Poseida Therapeutics, Inc. is sponsoring two U.S.-based clinical trials making use of PB technology to manufacture BCMA-specific CAR-T cells for patients with relapsed/refractory multiple myeloma (clinicaltrials.gov ID: NCT03288493) and prostate-specific, membrane antigen-specific CAR-T cells for patients with metastatic castration-resistant prostate cancer (clinicaltrials.gov ID: NCT04249947). Reports of the Phase-I BCMA-specific CAR-T cell trial show highly encouraging results, with significant efficacy, low rates of cytokine release syndrome (CRS), and neurotoxicity.^47^ Accordingly, a subsequent Phase-II study has begun with a planned sample number of 100 patients in an outpatient setting given the unique safety profile observed in Phase-I.^48^

Unfortunately, The CARTEL Study has recently produced unexpected serious adverse events. Following CAR-T cell infusion, two patients developed T cell lymphoma leading to the death of one of the patients.^49,50^ Analysis of the first lymphoma showed a high CAR copy number, but no insertion into typical oncogenes. A detailed analysis of the samples revealed transcriptional changes of many genes driven by the transgene promoter despite insulator sequences surrounding the transgene. However, these changes correlated with genomic copy number variations rather than with the PB vector insertion sites.^49^ Although the molecular events that led to these adverse events are currently unknown, the potential oncogenic activity of the PB system needs to be rigorously addressed.

### T cell modification through RNA transfection

The nonintegrating RNA platform offers multiple options to induce transient expression of immunoreceptors or other transgenes of interest in T cells or other immune cells such as NK cells. Here, we review the development of RNA-based CAR-T cells and the main clinical results obtained so far (Table 2).

**Table 2.** Clinical trials with reported data using RNA-electroporated or genome-edited CAR-T cells

RNA-Electroporated Autologous T cells						
Center	Disease	Target	Dose/Administration/Route of Delivery	Toxicity	Responses	Clinicaltrial.gov identifier and References
University of Pennsylvania	Triple negative breast cancer	cMet	Single intratumoral injection	No adverse effects greater than grade 1	CAR mRNA was detectable in blood and injected tumor tissues, IHC showed: tumor necrosis, loss of c-Met expression, intratumoral macrophage infiltration	NCT01837602 Ref.^63^
University of Pennsylvania	Refractory metastatic Pancreatic ductal adenocarcinoma	Mesothelin	Multiple infusions (three times weekly for 3 weeks)	No CRS or neurologic symptoms, no dose-limiting toxicities	2SD, antitumor activity as assessed by reduction in FDG uptake at PET CT imaging	NCT01897415 Ref.^62^
University of Pennsylvania	Solid tumors	Mesothelin	Repeated infusions of the CAR-T cells	No overt evidence of off-tumor on-target toxicity against normal tissues	Epitope spreading and antitumor activity were shown in both patients reported	NCT01355965 Ref.^54^
University of Pennsylvania	Refractory or relapsed Hodgkin lymphoma	CD19	IV, repeated infusions (up to six doses) after lymphodepleting regimen	No severe toxicities	Transient responses	NCT02277522 (adult) NCT02624258 (pediatric) Ref.^60^
University of Pennsylvania	Acute myeloid leukemia	CD123	Repeated infusions (up to six doses) of the CAR-T cells, with or without prior lymphodepleting regimen	Fever or CRS of varying severities, no severe toxicities	No antitumor effect	NCT02623582 Ref.^59^
University of Pennsylvania	Pancreatic adenocarcinoma	Mesothelin	Repeated infusions of the CAR-T cells	Anaphylaxis and cardiac arrest within minutes due to human anti-mouse immunoglobulin (Ig)G antibodies	Progressive disease	NCT01355965 Ref.^54^

Genome-Edited Allogeneic T Cells						
Center	Disease	Target	Gene editing method	Gene(s) knock out	Responses	Clinicaltrial.gov identifier
Institut de Recherches Internationales Servier	B cell acute lymphoblastic leukemia	CD19	TALEN	TCRAC and CD52	14 (67%) of 21 patients achieved a complete response or complete response with incomplete hematological recovery 28 days after infusion.	NCT02808442 NCT02746952 Ref.^66,86^
Chinese PLA General Hospital	Multiple solid tumor types	Mesothelin	CRISPR/Cas9	TCRAC and PD1	2 (13.3%) of 15 patients achieved a stable disease	NCT03545815 Ref.^79^
Xinqiao Hospital of Chongqing	T cell acute leukemia/lymphoma	CD7	CRISPR/Cas9	TCRAC and CD7	Initial results demonstrated that 4 (80%) of the patients displayed robust CAR-T cell expansion and achieved complete response	NCT04264078 Ref.^76^
Nanjing Bioheng Biotech Co., Ltd.	B cell acute lymphoblastic leukemia	CD19/CD22	CRISPR/Cas9	TRAC and CD52	5 (83.3%) of 6 patients achieved a complete response on day 28 after infusion	NCT04154709 Ref.^87^

CRS, cytokine release syndrome.

#### Concept and biology of RNA electroporation

RNA coding for proteins to enhance immune cell functions and phenotypic features can be a powerful tool to obtain therapeutic antitumor cell products. mRNA is usually *in vitro* transcribed and transfected using electroporation. There is extensive preclinical and clinical data showing the feasibility of RNA CAR electroporation into human T cells to endow them with tumor lytic functions both *in vitro* and *in vivo*.^51^ Moreover, a variety of RNA-based vectors have been optimized for T cell transfection, CAR expression, and RNA production.^52^

Permanent integration of the CAR transgene into the T cell genome can mediate long-term persistence of the transduced T cells *in vivo*, potentially triggering “on-target off-tumor toxicity” when targeting tumor-associated antigens also expressed at low levels in healthy tissues. RNA-based transfection results in transient expression of the CAR construct, as RNA is translated into CAR proteins and expressed on the cell surface for a maximum of 7 days.^53^ Thus, this technology offers the opportunity to evaluate the safety of previously noninvestigated scFvs or CAR constructs.

The downside of such short-lived electroporated T cells is the potential reduced antitumoral effects. However, RNA CAR-T cells can be administrated multiple times as a way to circumvent this potential drawback.^54^ Attempts to increase CAR expression durability have been pursued by modifying open reading frames, nucleotide capping, and poly (A) tail lengthening (reviewed in Pohl-Guimarães et al.^55^). However, while transgene expression may be improved, no increase in CAR expression stability was observed.

An important advantage of RNA CAR-T cells is the fast and cost-effective manufacturing that can be as short as 24 h when *in vitro* transcribed RNA encoding the CAR molecule is electroporated into resting T cells. These CAR-positive T cells can be readily infused 24 h postelectroporation, which is extremely attractive in the clinic, in comparison with the 10- to 14-day manufacturing time needed for virus- or transposon-engineered CAR-T cells.

Simultaneous expression of multiple factors is another desirable feature with the nonintegrating RNA platform. Simon *et al.* showed feasibility and improved antitumor function *in vitro* of T cells following electroporation of multiple mRNA encoding for either TCR/CARs, and siRNAs allowing significant knockdown of checkpoint molecules (programmed cell death 1 [PD-1], CTLA4).^56^ Other immune cells can also be used as effectors, such as NK cells electroporated with CAR-encoding RNA with even higher transfection efficiency and cytotoxicity in a CLL model.^57^

Preclinical evidence in murine and also in large animal models such as canines was very informative in that setting. Of note, mRNA electroporation has been used with canine T cells to treat CD20^+^ B cell high-grade dog lymphoma. Anti-canine CD20 RNA canine CAR-T cells were able to expand, secrete proinflammatory cytokines, and specifically lyse CD20^+^ canine tumor cells in preclinical studies.^58^ Next, in a first-in-canine study, Panjwani *et al.* used autologous anti-CD20 CAR-T cells to treat a dog with relapsed B cell lymphoma. This work provided proof-of-concept of safety and transient antitumor activity in an immunocompetent large animal model.^58^

#### Lessons learned from RNA-electroporated CAR-T cells in the clinic

RNA CAR-T cells have already been tested in the clinic in different tumor indications, including hematologic malignancies and solid tumors, and proved to be safe with a biological effect, but modest clinical impact.

##### Anti-CD123 RNA CAR-T cells

Short-term toxicity of serial infusions of transient CAR-T cells was assessed in heavily pretreated patients with recurrent/refractory AML using T cells electroporated with anti-CD123 CAR mRNA^59^ (NCT02623582). Five adult patients received multiple IV doses of “RNA CART123” with an optional lymphodepleting cyclophosphamide-based regimen. The primary objective was to assess the safety, with secondary objectives being evaluation of persistence and trafficking of RNA CART123 cells and also a reduction in blast percentage, response rate, overall survival, time to relapse, and percentage of subjects who subsequentially received allogeneic stem cell transplant (ASCT).

No overt toxicity was observed; all patients presented CRS manageable with anti IL-6 therapy. CAR-T cells modestly peaked in the peripheral blood, with no *in vivo* expansion. Investigators did not observe a decrease in CD123 expressing cells in the bone marrow, and all treated patients presented a disease progression. The clinical outcome of this trial stresses the importance of the quality of the T cells electroporated, which might have been suboptimal in this heavily pretreated patients' cohort (median number of prior lines of treatment was 4 and 4/7 patients had undergone prior ASCT). However, RNA CART123 were able to have a biological effect with CRS.

##### Anti-CD19 RNA CAR-T cells

Svoboda *et al.* reported a pilot trial using CD19 targeting CAR-Ts in patients with relapsed or refractory Hodgkin's lymphoma using nonviral RNA CART19 cells. Among the 4 treated patients, no severe toxicities were observed with transient responses achieved^60^ (NCT02277522).

##### Anti-mesothelin RNA CAR-T cells

Mesothelin is a cell surface tumor-associated antigen overexpressed in a variety of solid tumors (notably ovarian and pancreatic adenocarcinoma). Three trials reported the safety and efficacy of RNA encoding a mesothelin-targeting CAR in T cells. Maus *et al.* utilized mRNA coding for a CAR derived from a murine anti-mesothelin antibody with multiple infusions.^61^ One subject (out of four treated) developed anaphylaxis and cardiac arrest within minutes upon third infusion. This was the first report of anaphylaxis following CAR-electroporated human T cells, attributed to IgE antibodies directed against the CAR molecule. These results suggested immunogenicity of murine CARs and potential safety issue, but the role of repeated infusions is still not clear.

Using an *in vitro* transcribed mRNA encoding a 4–1BBz-based CAR in two patients, Beatty *et al.* showed safety with no of “off-tumor on-target” toxicity. These transiently persistent engineered cells migrated to primary and metastatic tumor sites. This work provided evidence of antitumor activity both clinically and biologically, including the induction of humoral epitope spreading after RNA CARTmeso cell infusion.^54^

More recent results from a Phase I trial in patients with pancreatic ductal adenocarcinoma (PDAC) treated with autologous anti-mesothelin RNA CAR-T cells confirmed these data. Six patients with refractory PDAC were included and patients received infusions three times weekly for three weeks. No CRS, neurotoxicity, nor dose limiting toxicities were observed, and interestingly, radiological responses in liver metastasis but not the primary tumor site were seen with also two disease stabilizations.^62^ Importantly, this set of clinical data shows antitumor activity in solid tumors refractory to all other standard therapies.

##### Anti-cMet RNA CAR-T cells

cMet is a surface antigen expressed in breast cancer, thyroid cancer, and nonsmall-cell lung cancer (NSCLC), among others. A Phase 0 trial investigated intratumoral administration of cMet targeting RNA CAR-T cells in patients with metastatic breast cancer with accessible cutaneous or lymph node metastases in a single intratumoral injection. The autologous T cell product was detected in both peripheral blood and the injected tumor site. Immunohistochemistry analysis performed in the resected injected tumors revealed extensive tumor necrosis, cellular debris, and tumor infiltration with immunosuppressive macrophages (with increased CD163/CD68 ratio) on the edges of tumor plane.

Median follow-up was 10 months in the six patients (four of six had triple negative breast cancer with dismal prognosis). Unfortunately, only one patient had a stable disease in this small cohort.^63^ These results indicate that while mRNA c-Met-targeted CAR-T cells were well tolerated and evoke an inflammatory response, this was insufficient to induce objective responses in patients.

Based on these encouraging clinical and biological results in multiple solid tumors, the field is awaiting results from other early-phase trials. For example, Descartes-11- an open-phase 2 trial is investigating an RNA CAR-T cell therapy in patients with newly diagnosed high-risk multiple myeloma with residual disease after induction therapy (NCT04436029).

## Genome Editing in CAR-T Cells

The discovery and rapid development of nucleases that can be engineered to recognize and cleave DNA at predetermined sites in the genome have given researchers the capacity to incorporate sequence-specific alterations using nonviral systems into a wide range of cell types and species.^64^ For this purpose, various designer nucleases, including zinc-finger nucleases (ZFNs), transcription activator-like effector nucleases (TALENs), and the clustered regulatory interspaced short palindromic repeat/associated nuclease protein 9 system (CRISPR/Cas9), have been utilized. In the field of CAR-T cells, genome editing has been used for knockout (KO) and knockin (KI) strategies to generate allogeneic universal “off-the-shelf” CAR-T cells, to KO inhibitory receptors or to KI the CAR gene or additional transgenes into desired loci.

### Mechanism of action: double-stranded breaks and mechanisms of DNA repair

TALENs, ZFNs, and the CRISPR/Cas9 system enable efficient and precise genetic modifications by inducing targeted DNA double-strand breaks (DSBs) that trigger two main mechanisms of repair.^65^ The predominant one, nonhomologous end-joining, mediates direct ligation of the broken DNA and can result in the insertion or deletion of mutations due to its error-prone nature. This yields a KO if the mutation is situated in a coding region of a gene and results in a frameshift. The other mechanism of repair is the homology-directed repair (HDR), which is less frequent, much slower, and mainly utilized during the late S- and G2-phases of the cell cycle (*i.e.*, when DNA replication is completed, and the sister chromatids are available to serve as repair templates). HDR enables site-specific transgene integration at the break site (KI) when using donor DNA sequences flanked by homology arms.

Briefly, TALENs are generated by fusing a TALE (TALE) DNA-binding domain to a DNA cleavage domain (catalytic domain of the bacterial *Fok*I nuclease). Because the TALEs can be designed to bind virtually any target DNA sequence, cuts at specific locations within the genome can be achieved when they are combined with the nuclease. At their origin, TALEs are proteins that are secreted by Xanthomonas bacteria via their type III secretion system when they infect plants. The DNA binding domain comprises a repeated and highly conserved 33–34 amino acid sequence that varies at the 12th and 13th positions (referred to as the repeat variable diresidue; RVD). The RVDs are highly variable and govern specific nucleotide recognition.

TALENs have been used to develop universal allogeneic T cells,^66^ but the extensive protein engineering needed to transition between gene targets has limited their broad use. Moreover, their large size makes them more difficult to deliver than ZFNs. 

ZFN domains are relatively small protein motifs comprising multiple finger-like protrusions that contact their target molecule (DNA, RNA, protein, and/or lipid substrates). Their binding properties depend on the amino acid sequence of the finger domains, the linker between fingers, the number of fingers, and their higher order structure. Tandem repeats of engineered zinc fingers can be used to target desired DNA sequences, and fusion of the ZF domain to the *Fok*I DNA cleavage domain allows site-directed cuts in the genome. The C_2_H_2_ ZFN, for which each finger recognizes 3–4 bp of DNA via a single α-helix, was originally discovered in Xenopus and is the most common DNA binding motif in all metazoa.

While ZFNs have been utilized in cancer immunotherapy trials, they have mostly been used to target CCR5 and CXCR4 to abrogate HIV infection of T cells. Like TALENs, a new ZFN has to be engineered, selected, and optimized for each new DNA target site. Finally, CRISPR/Cas9, which requires minimal alteration relative to TALENs and ZFNs to be directed against new target sites within the genome, is emerging as a more favorable approach for T cell engineering.

### CRISPR/Cas9 technology takes the lead

Because of its simplicity, flexibility, multiplex genome editing capacity, and specificity, CRISPR/Cas9 is poised to revolutionize gene therapy and will likely become a critical tool for the engineering of T cells for cancer immunotherapy. In nature, the CRISPR/Cas system, of which there are 6 types and 29 subtypes, is a prokaryotic-acquired immunity mechanism enabling cleavage of invading nucleic acids during bacteriophage reinfection.^67^

Cas9, which belongs to the Class 2, type II CRISPR system and originates from *Streptococcus pyogenes*, was the first to be reprogrammed for genome editing of mammalian cells and is currently the one most widely used for T cell engineering.^68,69^ CRISPR/Cas9 is a two-component system consisting of a single guide (g)RNA and the Cas9 endonuclease that creates DSBs in the genome.^70^ Briefly, the gRNA complexes with Cas9 and is specifically designed for complementary base pairing to the site of interest in the genome. The CRISPR system can be targeted to virtually any region in the genome to create a DSB provided that it is adjacent to a 3′ protospacer-adjacent motif (PAM) sequence (NGG for Cas9); the DNA break occurs 3–4 bp upstream of the PAM sequence.

Early attempts at CRISPR/Cas9 editing of primary human T cells included viral vectors or plasmids for Cas9 and gRNA expression, but low targeting efficiency and high toxicity were observed. In addition, the risk of unwanted genetic mutations and immunogenicity pose safety concerns for clinical translation.

More recently, a ribonucleoprotein (RNP)-based approach comprising complexes of recombinant Cas9 protein and synthetic gRNA has demonstrated high efficiency.^71^ While it is possible to generate gRNA via *in vitro* transcription, the presence of 5′ triphosphate single-stranded RNA (ssRNA) can activate a type I interferon-mediated immune response.^72^ To avoid this undesirable effect, the use of commercially available synthetic gRNAs is highly recommended. Different web tools can be used to select target sites in the genome and design gRNA. Notably, high-fidelity variants of Cas9, such as Spyfi, have been developed and reported to have reduced off-target cutting in the genome and are also commercially available for clinical-grade production of cellular therapies.

### Endonuclease-mediated KO in CAR-T cells

Genome editing can bypass issues related to the inability to produce sufficient CAR-T cells from autologous sources via the generation of allogeneic universal “off-the-shelf” CAR-T cells by KO of components of the TCR and the HLA, such as the TCRα and TCRβ constant chains (*TRAC* and *TRBC*) and beta-2 microglobulin (*B2M*), respectively.^73^ Simultaneous editing of TCR genes reduces the risk of GVHD in the allogeneic setting.

A first-in-human clinical trial using gene-edited universal CAR-T cells highlighted the feasibility and potency of this approach (Table 2). Using TALEN-mediated genome editing in combination with lentiviral transduction and magnetic bead depletion of residual TCRαβ T cells, stringent depletion of TCRαβ was achieved (>99% of CAR-T cells were depleted of endogenous TCR). Treatment of two infants with B-ALL with this universal CAR-T cells showed successful induction of molecular remission and T cell persistence without significant GVHD.^66^

Several other clinical trials using gene-edited allogeneic CAR-T cells are under way, and some early results have been presented in conferences (Table 2). For example, the CALM (NCT02746952) and PALL (NCT02808442) studies presented pooled data with universal CD19-CAR-T cells for the treatment of adult and pediatric ALL patients. Encouraging clinical responses were observed (88% of CR or CRi by day 28 or 42 with a peak of CAR-T cell expansion observed in 72% of patients) with an acceptable safety profile. While these results are promising, more patients and a longer follow-up are needed to evaluate the safety and efficacy of universal CAR-T cells compared with their autologous counterparts.

Genome-edited CAR-T cells are also being tested in patients with T cell malignancies. One of the major limitations of the development of CAR-T cell therapies for T cell tumors is that normal and malignant T cells share the expression of most of the targetable antigens, which can result in T cell fratricide during CAR-T manufacturing. This fratricide can be abrogated, for example, by KO of *CD7* or *CD5* before transduction with CARs targeting these respective antigens.^74,75^ Such genome editing to circumvent fratricide is currently being evaluated in patients with hematologic malignancies (NCT04264078) and treated with CD7-redirected CAR-T cells with disrupted *CD7* and *TRAC* genes. Initial results demonstrated that 80% of the patients displayed robust CAR-T cell expansion and achieved complete response with an acceptable safety profile.^76^ Hence, this KO strategy can be applied in future clinical trials evaluating the efficacy of CAR-T cells targeting solid tumor antigens that are coexpressed on T cells.

Also, it has been widely demonstrated that endonuclease-mediated KO of genes encoding various regulatory receptors or molecules significantly boosts the therapeutic efficacy of tumor-directed T cells in preclinical studies, either by directly promoting T cell function or by helping them to overcome barriers in the TME.^71^ Since 2020, the safety and feasibility of using the CRISPR/Cas9 system to engineer antitumor T cell products for clinical use were reported in three independent trials, although only one of them used CAR-engineered T cells.

Stadtmauer *et al.*^77^ treated patients with refractory cancer with T cells in which CRISPR/Cas9 was used to disrupt 3 genes, *TRAC*, *TRBC*, and *PDCD1*, and a viral vector was used to introduce a tumor-directed TCR (NCT03399448). The treatment was well tolerated and there was durable engraftment of genome-edited cells during the study. In addition, Lu *et al.*^78^ safely treated 12 NSCLC patients with PD-1 gene-edited bulk autologous T cells (NCT02793856). Finally, a clinical trial that has been conducted recently demonstrated the feasibility and safety of CRISPR-engineered (*PDCD1* and *TRAC* KO) anti-mesothelin CAR-T cells for the treatment of multiple solid tumor types.^79^

While initial results are promising, long-term follow-up, dose escalation, and the treatment of a greater number of patients are required to fully evaluate the potential of CRISPR/Cas9-engineered T cells.

### Endonuclease-mediated KI in CAR-T cells

Genome editing techniques can be readily adapted to KI new transgenes into selected endogenous loci by delivering a donor DNA that encodes the transgene of interest flanked by homology arms specific to the gene target. This strategy allows the expression of the transgene under the natural promoter of the targeted gene, enabling physiological transgene regulation.

In a first approach in the field of CAR-T cells, donor DNA encoding for a CAR was introduced in the endogenous TCR locus using an AAV-6 following CRISPR/Cas9-mediated HDR. Using this approach, targeted integration of CAR transgene into the TRAC gene locus led to enhanced T cell activity by promoting optimal CAR expression and alleviating T cell exhaustion following repeated exposure to antigen.^80^ Disruption of *TRAC* post-CAR integration can further generate allogeneic universal CAR-T cells. Of note, apart from the native TCR locus, genes encoding for immune checkpoint molecules, such as *PDCD1*, are also good candidates for transgene integration sites and can lead to concomitant KO of PD-1.^81^

Because the generation and testing of viral vectors, such as AAV-6, can be lengthy and expensive, especially under GMP conditions, nonviral methods to deliver the DNA template have also been tested. A major limitation to deliver donor DNA into T cells is that introduction of large linear double-stranded DNA (dsDNA) sequences is toxic at high concentrations. Despite this, recent reports demonstrated that coelectroporation of CRISPR/Cas9 RNP complexes and long linear dsDNA templates (created using standard PCR amplification) or nanoplasmid DNA is a feasible strategy to KI genes in the T cell genome.^82,83^

## Concluding Remarks and Future Directions

Thanks to our improved understanding of T cell biology, together with extraordinary advances in genetic engineering and cell manufacturing, it is possible to genetically modify a patient's own T cells with desired tumor specificity and enhanced function. Second-generation CAR-T cells generated using γ-retroviruses or lentiviruses achieved clinical approval in 2017, and it is expected that additional CAR-T cell therapies will reach the global marked in the coming years.

Notably, after the treatment of hundreds if not thousands of patients around the globe with retroviral- and lentiviral-modified T cells, there have been no reports of confirmed positive RCR/L results in clinical vector lots, infusion products, or patients treated with T cell therapies. Based on these robust safety data, it has been suggested that the current guidelines for CAR-T cell therapy product testing and long-term patient monitoring should be revised to facilitate less cumbersome development and translation of T cell therapies.^21^ Similarly, no T cell transformation has been reported in patients treated with T cells genetically modified with viral vectors, indicating that the use of currently used viral vectors presents a favorable safety profile.

However, recent reports describing the first two cases of malignant lymphoma derived from PB-modified CAR-T cells emphasize the need for caution and regular monitoring of treated patients, in particular as engineering tools newly reach the clinic.

A potential alternative to integrative vectors that is under current preclinical investigation is nonintegrating episomal DNA nanovectors, such as the nano-S/MARt (nS/MARt).^11^ A manufacturing protocol to produce clinical-grade CAR-T cells using this technology has been developed, offering an easy, simple, and versatile alternative to the widely used viral vectors. While these vectors may avoid the risk of insertional mutagenesis by randomly integrating vectors, therapeutic efficacy of CAR-T cells engineered with this type of vector awaits clinical validation.

Early experience with second-generation CAR-T cells revealed numerous obstacles faced to unleash their full therapeutic potential, especially in the context of solid tumors. Considering heterogeneity of antigen expression and tumor escape mechanism through antigen loss, having a platform that would allow for the testing of CAR-T cells against multiple specificities could greatly advance the field. Moreover, it is likely that CAR-T cells will require further genetic modification to overcome the immunosuppression posed by the TME, as well as to circumvent tumor escape. To date, no clinical trial has reported results of combination of multivalent or next-generation CAR-T cells for the treatment of cancer. Hence, it is difficult to extrapolate on the potential toxicity of such combinations.

The electroporation of RNA encoding CARs or/and additional transgenes may help to advance next-generation approaches to first-in-human clinical trials due to the versatility to encode for different proteins and favorable safety profile. While no significant therapeutic effects have been observed for RNA-modified CAR-T cells, this approach is still very actively pursued. Multiple manufacturing parameters will need to be compared as there are a variety of methods to engineer T cells using *in vitro* transcribed RNAs. In fact, GMP-grade electroporation devices, alternative transfection methods (liposomes, nanoparticles), time points of transfection during T cell expansion, cytokine cocktails, or immune cells used (peripheral blood mononuclear cell, CD3-purified T cells, NKs) are a few of these parameters with a role to play in safety and effectiveness. However, while manufacturing of RNA is cheaper than viral vectors, the requirement of several doses of CAR-T cells per patient is a key limitation for the widespread use of this technology.

Genome editing by CRISPR/Cas9 holds great promise to advance the field of CAR-therapy due to its versatility and speed at which new targets for KO or KI can be evaluated in T cells due to the ease of generating gRNA. It is critical that this powerful technology advances carefully in the clinic as in the first-in-human studies for engineered T cells and that there is long-term follow-up of patients. The first CRISPR/Cas9 clinical trials comprising engineered T cells for cancer treatment have further demonstrated the feasibility to combine different engineering tools and this will likely be a strategy used frequently in the clinic in the future.

Finally, it is worth noting that *in situ* genetic reprogramming of T cells using lentiviral vectors or AAV carrying the CAR gene is being explored as an alternative to *ex vivo* T cell engineering^.84,85^ By direct injection of the gene vector, T cells can be genetically modified to express the CAR within the host. This strategy has been proven to be feasible in preclinical settings and could be an alternative to the highly demanding and resource-intensive currently used CAR-T manufacturing protocols. However, further studies are required before this strategy can be translated to the clinic.
